# AMPK activation by hepatitis E virus infection inhibits viral replication through attenuation of autophagosomes and promotion of innate immunity

**DOI:** 10.1007/s00018-025-05634-8

**Published:** 2025-03-13

**Authors:** Chunling Wang, Xiaoman Liu, Yao Zhao, Shumin Liao, Jiayue Zhang, Yanhong Huang, Yue Shi, Liang Li, Qiuwei Pan, Jian Wu, Yijin Wang

**Affiliations:** 1 Department of Pharmacology, Joint Laboratory of Guangdong-Hong Kong Universities for Vascular Homeostasis and Diseases, School of Medicine, Southern University of Science and Technology, Shenzhen, 518055 China; 2 Institute of Reproductive Health/NHC Key Laboratory of Birth Defects Prevention, Henan Academy of Innovations in Medical Science, Zhengzhou, China; 3 School of Pharmacy, Jiangsu Food & Pharmaceutical Science College, Huaian, Jiangsu China; 4 Department of Gastroenterology and Hepatology, Erasmus MC-University Medical Center, Rotterdam, 3015CE The Netherlands; 5 Department of Clinical Laboratory, The Affiliated Suzhou Hospital of Nnjing Medical University, Suzhou Municipal Hospital, Gusu School, Nanjing Medical University, 242 Guangji Rd, Suzhou, Jiangsu, 215008 China

**Keywords:** Viral infection, Metabolism, Antiviral medication, TBK1

## Abstract

Hepatitis E virus (HEV) infection is generally asymptomatic or leads to acute and self-limiting hepatitis. The mechanisms orchestrating such an infection course remain to be elucidated. AMP-activated protein kinase (AMPK) is a pivotal cellular sensor for maintaining metabolic homeostasis. Here, we show that AMPK is activated in response to HEV infection and is associated with mitochondrial damage and ATP deficiency. AMPK activation, in turn, inhibits HEV replication. Mechanistic studies reveal that AMPK activation triggers the expression of interferon (IFN)-stimulated genes that possess antiviral properties. In parallel, AMPK inhibits autophagosome accumulation to exert antiviral effects. Interestingly, AMPK activation also suppresses the inflammatory response triggered by HEV infection. Consistently, AMPK activation simultaneously exerts anti-inflammatory and antiviral effects in a coculture system of HEV-infected liver cells with macrophages. These findings pave the way for the development of AMPK-targeted therapeutics to treat hepatitis E.

**Supplementary Information:**

The online version contains supplementary material available at 10.1007/s00018-025-05634-8.

## Introduction

Hepatitis E virus (HEV) is a nonenveloped single-stranded RNA virus with a 7.2 kb genome that encodes three proteins: ORF 1, 2 and 3. There are eight genotypes (GTs) of HEV, among which GT 1, 2, 3, 4 and 7 can infect humans [1]. HEV infection is usually asymptomatic and self-limiting in healthy individuals, but it can cause fulminant hepatitis and even liver failure, with high mortality in pregnant women or patients with underlying liver diseases [2]. Proinflammatory cytokines, such as IFN-γ, TNF-ɑ, IL-10 and IL-18, have been reported to be associated with adverse outcomes in hepatitis E patients [3, 4]. GT3 or GT4 HEV infection in organ transplant recipients receiving immunosuppressive drugs is associated with a high risk of developing chronic hepatitis E, which can rapidly progress to liver fibrosis and cirrhosis [5, 6]. However, there is no FDA-approved anti-HEV medication available yet, despite the use of interferon-alpha (IFN-ɑ), ribavirin, or a combination of these drugs being used occasionally as off-label treatments. Therefore, the development of effective antiviral treatments through the identification of host therapeutic targets that respond to viral infection is urgently needed.

As a cellular energy sensor, adenosine monophosphate-activated protein kinase (AMPK) is generally activated during metabolic stress by increasing AMP/ATP or ADP/ATP ratios, leading to a switch of cells from anabolic to catabolic processes to maintain the cellular energy balance [7]. AMPK is a heterotrimeric complex of a catalytic α-subunit and two regulatory β- and γ-subunits [8]. AMPK is active only after phosphorylation of the α subunit at a threonine residue within the kinase domain (T172) by upstream kinases. In the context of viral infection, AMPK appears to perform virus-associated metabolic remodeling activities that play an adaptive role, either in promoting or inhibiting viral replication [9, 10]. However, the role of AMPK in HEV replication is still unclear.

The antiviral innate immune response and inflammatory response are two conspicuous lines corresponding to cellular infection or stress. The former largely relies on pattern recognition receptors (PRRs) that monitor and detect viral infection, followed by the activation of interferon (IFN) signaling and the expression of hundreds of interferon-stimulated genes (ISGs) [11–13]. The latter triggers the maturation of proinflammatory cytokines such as interleukin-1β to control viral infection and/or worsen disease progression by inducing inflammatory injury in target organs. Notably AMPK activation has been reported to increase the expression of IFNs and ISGs during viral infection [10, 14]. We previously demonstrated that HEV infection caused an IFN response in experimental models and the livers of acute HEV patients [15]. These studies suggest AMPK may play an important role in regulating innate immunity during HEV infection. Moreover, increasing evidence indicates that AMPK modulates inflammation [16, 17]. For example, activation of AMPK reduces the production of proinflammatory cytokines, including TNF-α, after Toll-like receptor 4 (TLR4) stimulation in neutrophils and macrophages [16, 18].

Autophagy is a process in which cytosolic components are degraded in lysosomes [19]. The accumulation of autophagosomes is generally associated with the initiation of autophagy and/or autophagic degradation. Autophagic flux is marked by the conversion of microtubule-associated protein 1 A/1B-light chain 3 (LC3-I) to LC3-II, which binds to p62/SQSTM1 to target cargo for degradation. Owing to its important role in cellular homeostasis, autophagy is an important cellular factor that many viruses utilize for replication [20–22]. AMPK is also a key regulator of autophagy in response to various stresses [23]. It activates autophagy by phosphorylating ULK1, inhibiting mTOR, and regulating PIK3C3/VPS34 complexes [22, 24, 25]. Whether AMPK is linked during HEV infection to modulate the autophagic process and how it, in turn, regulates viral replication is of interest.

Currently, the underlying molecular mechanism by which AMPK mediates HEV replication remains to be elucidated. We investigate the significance of AMPK during HEV infection using HEV acute and chronic infection models and the HEV subgenomic replicon models.

## Materials and methods

### Cell lines and human organoids

Huh7 cells, HepG2 cells, THP-1 cells, and HEK293T cells were obtained from the American Type Culture Collection (ATCC) and periodically tested for mycoplasma contamination. The information on the cells can be checked via the ATCC website. Huh7, HepG2 and HEK293T cells were cultured in Dulbecco’s modified eagle medium (DMEM) supplemented with 10% fetal bovine serum (FBS), 100 IU/mL penicillin and 100 mg/mL streptomycin. All the cells were maintained in a humidified atmosphere at 37 °C and 5% CO2. The human monocytic cell line (THP-1) was cultured in RPMI 1640 medium supplemented with 10% (v/v) inactivated FBS, 100 IU/mL penicillin and 100 mg/mL streptomycin. For macrophage differentiation, THP-1 cells were treated with 30 ng/mL phorbol 12-myristate 13-acetate (PMA) at 37 °C for 48 h. Then, the cells were cultured for another 24 h without PMA. Human organoids were cultured in advanced DMEM/F12 supplemented with 1% penicillin/streptomycin, 1 M HEPES, 200 mM ultraglutamine, 1% (v/v) N2, 2% (v/v) B27, 1 mM N-acetylcysteine, 10 mM nicotinamide, 5 µM A83.01, 10 µM forskolin, 10 nM gastrin, epidermal growth factor (EGF) (50 ng/ml), 10% (v/v) R-spondin-1 (conditioned medium), fibroblast growth factor 10 (FGF10) (100 ng/ml), hepatocyte growth factor (HGF) (25 ng/ml), and 10 µM Y27632. To ensure quality (genetically stable) and replicability (cryopreservation), early-passage organoids (up to 15 passages) were used in this study.

### HEV models

Chronic HEV-infected Huh7 cells (p6 cells) and HEV subgenomic replicon models (p6-Luc) were established as previously described [2]. A plasmid construct containing the full-length HEV genome (Kernow-C1 p6 clone, GenBank Accession Number JQ679013) and a construct containing a subgenomic HEV sequence coupled with a Gaussia luciferase reporter gene were used to generate HEV genomic RNA via the Ambion mMESSAGE nMACHINE in vitro RNA transcription kit. Huh7 cells and organoids were electroporated with full-length HEV genome to generate infection models. Huh7 cells were electroporated with subgenomic RNA to generate replication models. For the acute HEV infection model, human hepatic Huh7 and HepG2 cells were inoculated with infectious HEV particles. Infectious HEV particles were produced from p6 cells. Briefly, many p6 cells were collected and subjected to repeated freeze-thaw cycles to lyse the cells. The lysed cell suspension was then centrifuged at 1000–2000 rpm to remove cell debris. The supernatant was collected and subjected to ultracentrifugation at 22,000–40,000 rpm to pellet the HEV particles. The resulting HEV pellet was resuspended in an appropriate buffer to obtain infectious HEV particles.

### Immunofluorescence

The cells were subsequently grown on coverslips and treated as indicated. Then, the cells were fixed in 4% paraformaldehyde at room temperature for 15 min. After being washed three times with PBS, the cells were permeabilized with PBS supplemented with 0.1% Triton X-100 for 10 min, washed three times with PBS, and finally blocked with 5% goat serum for 1 h. The cells were then incubated with primary antibodies overnight at 4 °C, followed by incubation with the corresponding secondary antibodies for 1 h. After being washed three times, the cells were incubated with Antifade Mounting Medium with DAPI (P0126, Beyotime) for 5 min and then washed three times with PBS. Finally, the cells were analyzed via a laser-scanning confocal microscope (LSM 900) and quantitatively evaluated via ImageJ software.

To visualize of autophagosomes, Huh7 cells were transduced with a lentiviral vector expressing the mCherry-GFP-LC3 fusion protein. After 48 h of lentiviral infection, these cells were used for the indicated experiments. Following the corresponding experimental treatment, the cells were fixed in 4% formaldehyde and permeabilized in 0.5% TritonX-100. The cell nuclei were stained with DAPI dye, and the slides were imaged via a laser-scanning confocal microscope (LSM 900). Images were quantified via ImageJ software v10.

### Luciferase reporter assay

P6-luc cells were seeded in 96-well plates and treated with AICAR for 48 h. The activity of Gaussia luciferase was measured via a Gaussia-Lumi™ Gaussia Luciferase Reporter Gene Assay Kit to quantify viral replication. Gaussia luciferase activity was quantified with a LumiStar Optima luminescence counter (Gen5, BioTek, USA).

### Detection of MMP and ROS by flow cytometry

The cells were collected from the 6-well plates and then incubated with various dyes: 100 nM TMRE for 45 min at 37℃ to measure mitochondrial membrane potential (MMP) and 10 mM DCFH-DA for 20 min at 37℃ to measure reactive oxygen species (ROS). All samples were analyzed on a BD LSRII flow cytometer via FlowJo Software.

### Transmission electron microscopy

To detect the effect of HEV on mitochondria, Huh7 cells were seeded in 10-cm dishes and infected with HEV for 48 h. The cells were collected and fixed overnight in a fixative buffer with 2% formaldehyde plus 2% glutaraldehyde. The cell pellets were washed, dehydrated, and embedded according to the standard protocol. Ultrathin Sect. (90 nm) were cut, mounted on nickel grids, and stained with uranyl acetate-lead citrate. The sections were observed, and digital images with scale bars were collected via a HitachiH-7600 transmission electron microscope (Hitachi High Technology, USA).

### RNA-sequencing (RNA-seq) assays

Total RNA was extracted from cells using TRIzol reagent (Invitrogen) according to the manufacturer’s instructions. The RNA was treated with DNase I for 30 min at 37 °C to remove residual DNA. Qualified samples were used for library construction. Paired-end libraries were prepared via an ABclonal mRNA-seq Lib Prep Kit (ABclonal, China) following the manufacturer’s instructions. Library quality was assessed on an Agilent Bioanalyzer 4150 system. Finally, the library preparations were sequenced on an Illumina NovaSeq 6000 (or MGISEQ-T7) and 150 bp paired-end reads were generated. The data generated from the Illumina (or BGI) platform were used for bioinformatics analysis.

### Metabolite profiling

The cells were infected with or without HEV for 48 h. Metabolites were extracted in ice-cold methanol/acetonitrile/water (2:2:1, v/v) extraction solvent. Endogenous metabolite profiles were obtained by LC-MS methods using a UHPLC (1290 Infinity LC, Agilent Technologies) coupled to a QTRAP MS (6500+, Sciex). MultiQuant or Analyst was used for quantitative data processing.

### Coimmunoprecipitation (co-IP)

Huh7 cells in ten 15 cm dishes were transfected with the indicated expression plasmids and treated with HEV particles for 48 h. Then, the cells were collected and lysed in ice-cold lysis buffer with a proteinase inhibitor cocktail and a phosphatase inhibitor cocktail on ice for 30 min. The insoluble fraction of the cell lysates was removed by centrifugation at 10,000 × g and 4 °C for 15 min. The supernatants were collected and incubated at 4 °C with the indicated primary antibody overnight and then with protein A/G-agarose beads for 4 h. After extensive washes, the immunoprecipitates were resuspended in SDS sample buffer and boiled for 5 min for Western blotting.

### Coculture of macrophages with Huh7 cells harboring HEV

THP-1 cells were treated with 30 ng/ml PMA at 37 °C for 48 h. Then, the cells were cultured for another 24 h without PMA. A coculture of THP-1 macrophages with p6 cells was established at a ratio of 1:4, mimicking the relative percentages of these cell populations in the human liver.

### Quantification and statistical analysis

The statistical significance was assessed via unpaired two-tailed Student’s *t*-tests using SPSS 26.0 software. All the results are presented as the mean ± SEM. P values of less than 0.05 (single asterisks in the figures) were considered statistically significant, whereas *P*-values of less than 0.01 (double asterisks) and 0.001 (triple asterisks) were considered highly significant.

## Results

### HEV infection activated AMPK phosphorylation

**Fig. 1:**
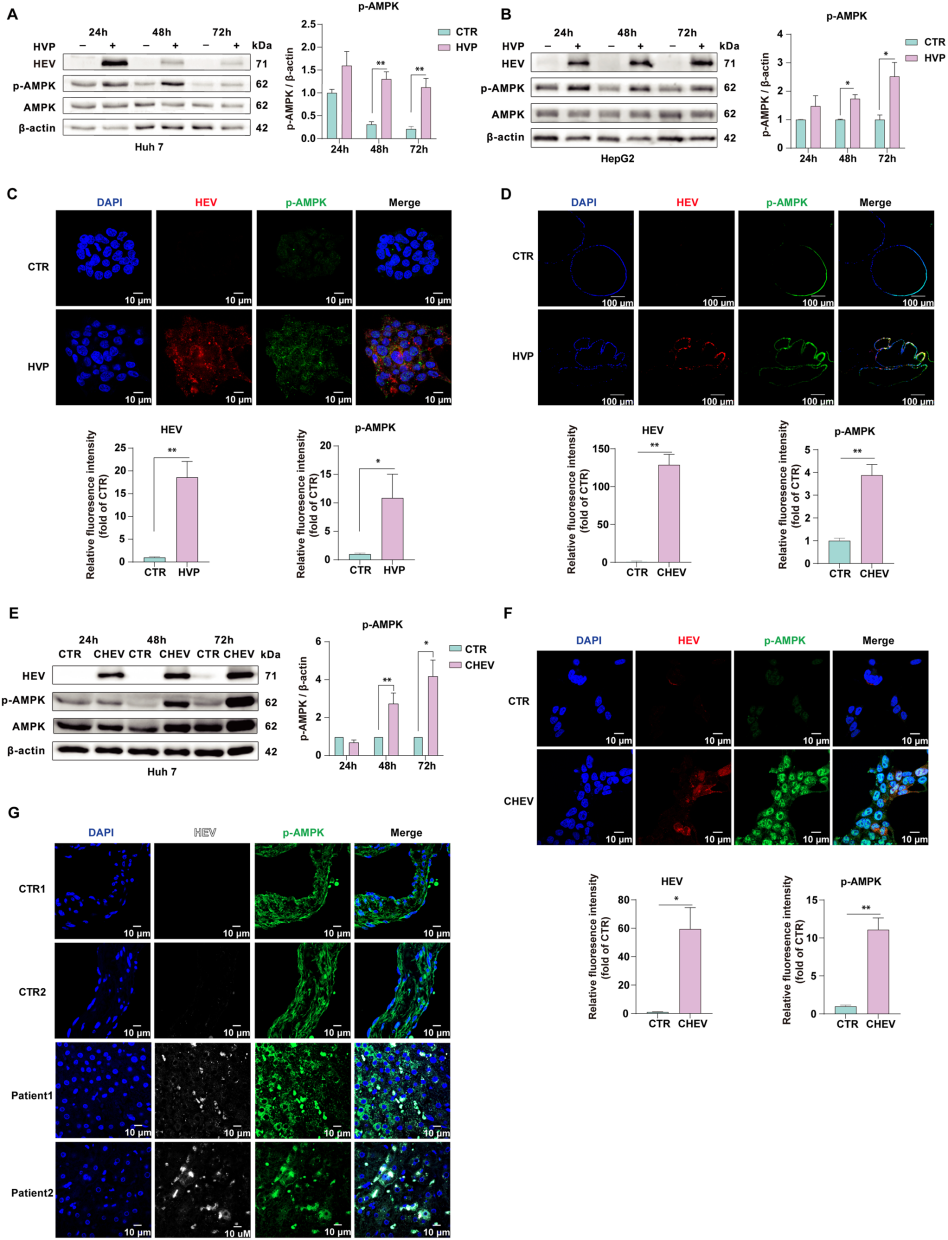
HEV infection activated AMPK phosphorylation. The protein levels of HEV (ORF2), p-AMPK and AMPK were quantified in Huh7 cells (**A**) and HepG2 cells (**B**) inoculated with HEV particles. Immunofluorescence staining for HEV (ORF2, red) and p-AMPK (green) was performed in Huh7 cells (**C**) inoculated with HEV particles for 48 h, as well as in organoids (**D**) cultured from adult human liver and transfected with HEV RNA on the 10th day following electroporation. (**E**) The protein levels of HEV, p-AMPK and AMPK and (**F**) immunofluorescence staining of HEV (ORF2, red) and p-AMPK (green) were quantified in Huh7 cells stably harboring the HEV RNA genome. (**G**) Liver tissues were subjected to immunostaining for HEV (ORF2, white) and p-AMPK (green). The data represent at least three experiments and are presented as the mean ± SEM. *P < 0.05; **P < 0.01; ***P < 0.001

We first assessed the expression and activation of AMPK in different HEV models. AMPK is active only after phosphorylation of the α subunit at a threonine residue within the kinase domain (T172) by upstream kinases. Naïve Huh7 cells inoculated with cell- culture-produced infectious HEV particles did not affect the expression of AMPKα or its upstream regulatory kinase genes *CAMKK1*, *CAMKK2* and *STK11* at the transcriptional level at 24 h post-infection (Fig. S1A). Immunoblot analysis revealed that successful HEV infection, as indicated by the presence of the HEV capsid protein (ORF2), in both the Huh7 and HepG2 cell lines facilitated the phosphorylation of AMPKα (Thr172) at 24 h, 48 h and 72 h (Fig. 1A and B), with no change in the total level of AMPK. Accordingly, positive p-AMPK was observed in HEV-infected Huh7 cells with concomitant HEV particles via confocal microscopy (Fig. 1C). Considering the clinical relevance of direct infection on bile duct epithelia (cholangiocytes) in patients with chronic hepatitis E [26], we cultured organoids from the adult human liver. We next profiled the expression of p-AMPK in organoids transfected with full-length GT3 (Kernow-C1 p6 strain) HEV RNA. We observed an increase in p-AMPK on day 10 after electroporation via immunofluorescence (Fig. 1D). These data demonstrated that AMPKα was potently activated in response to HEV infection in acute infection models. To determine whether the HEV structural protein ORF2 is responsible for modulating AMPK, Huh7 cells were transfected with a plasmid expressing HEV ORF2 for 24, 48–72 h, after which AMPK expression and activation were measured. We found that Huh7 cells expressing the HEV ORF2 protein presented increased levels of *PRKAA1*, *PRKAA2*, and their upstream regulators (*CAMKK1*, *CAMKK2* and *STK11*) at 48 h after transfection (Fig. S2A). Interestingly, in both Huh7 and HepG2 cells, HEV ORF2 transfection did not affect p-AMPK levels at any of the three-time points observed (Fig. S2B-S2C), indicating that ORF2 does not seem to be a primary element in AMPK activation.

To further investigate AMPK activation in a chronic HEV infection model, we examined the gene transcription and activity of AMPKα in Huh7 cells stably harboring HEV generated by transfecting full-length HEV gRNA for a long period. Consistent with the findings in acute HEV-infected cells, the transcriptional levels of AMPKα and its upstream regulators were not affected (Fig. S1B). However, AMPKα phosphorylation levels were significantly greater in HEV-infected cells than in noninfected cells (Fig. 1E and F). These findings show that in cells acutely infected with HEV or harboring HEV chronically, the phosphorylation of AMPK is dramatically triggered.

Finally, we evaluated the level of p-AMPK in hepatitis E patients who were admitted to the hospital because of complications. Two liver biopsies from patients diagnosed with acute hepatitis E were immunofluorescently stained with anti- HEV ORF2 and anti-p-AMPK antibodies. Two biopsies from a hepatic hemangioma patient without HEV infection served as a control. P-AMPK was markedly increased in hepatitis E patients (Fig. 1G).

### HEV infection reduced ATP levels caused by mitochondrial impairment

**Fig. 2:**
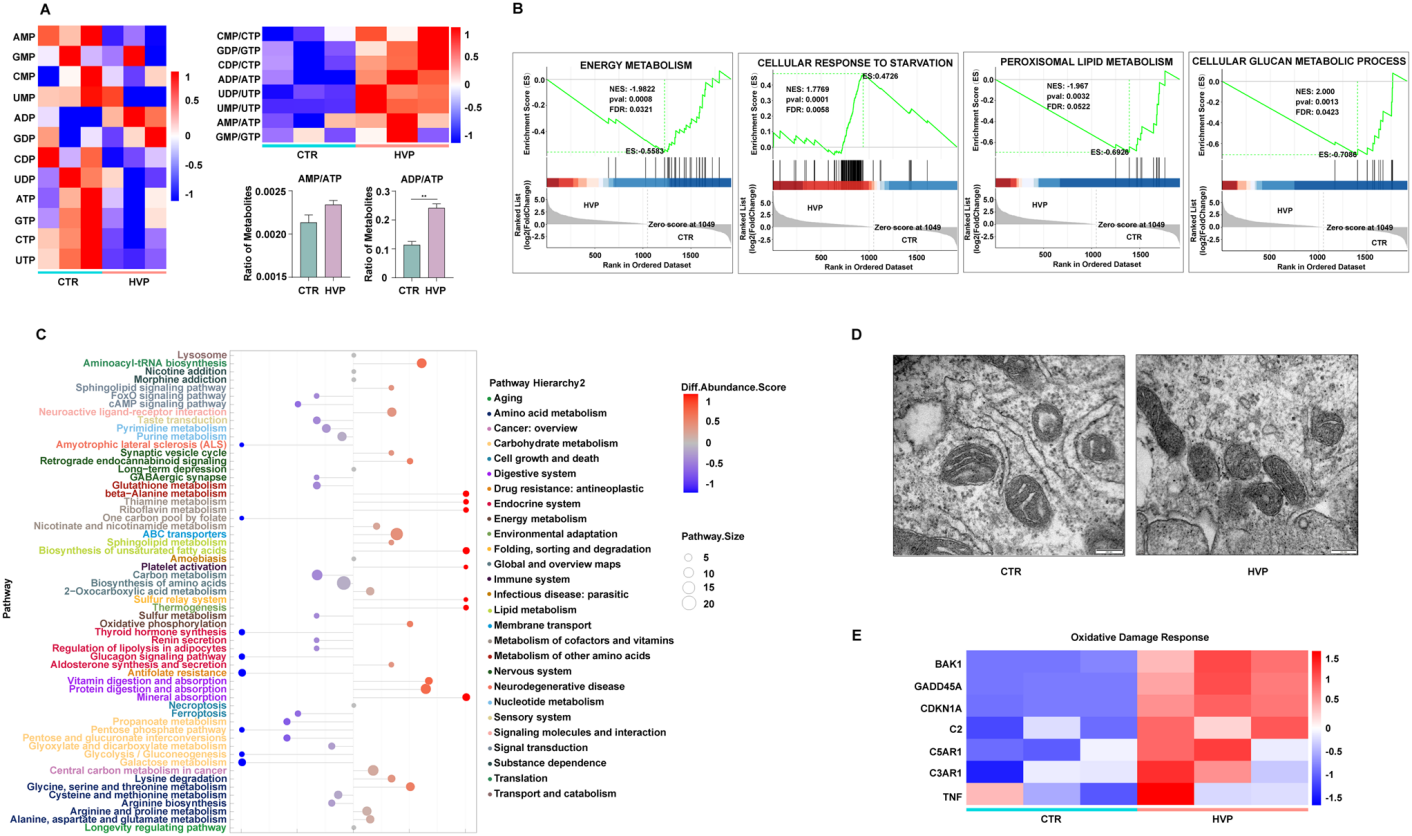
HEV infection reduced ATP levels caused by mitochondrial impairment (**A**) Nucleotide alterations in Huh7 cells compared with acute HEV-infected Huh7 cells. Gene set enrichment analysis (**B**) and a differentially abundant metabolite differential abundance score plot (**C**) of Huh7 cells with or without acute HEV infection revealed significant alterations in all metabolic pathways, including lipid, glucan, and energy pathways, in both omics analyses. (**D**) Transmission electron micrographs of mitochondria in acute HEV-infected cells. All images are at the same magnification. (**E**) Heatmap showing that NAD metabolism, mitochondrial damage, and oxidative damage response were enhanced upon acute HEV infection. All the heatmaps are generated after Z score normalization. The data represent at least three experiments and are presented as the mean ± SEM. *P < 0.05; **P < 0.01; ***P < 0.001

AMPK activation depends on nutrient deprivation, calcium fluctuations, and increased ADP/ATP ratios in concert with AMP/ATP. To study whether the metabolic changes upon HEV infection are responsible for the phosphorylated activation of AMPK, we compared energy metabolism via a high-throughput targeted mass spectrometry approach and gene expression via RNA sequencing (GenBank Accession Number: GSE262469) in acute HEV-infected and mock-infected cells. This analysis revealed that acute HEV infection resulted in reduced levels of high-energy phosphate molecules (UTP, ATP, GTP, CTP). These perturbations increased the ADP/ATP ratio (Fig. 2A), suggesting a reduced capacity for mitochondrial oxidative metabolism, which ultimately reduced energy generation and activated AMPK. Pathway enrichment showed marked alterations in all metabolic pathways, including lipid, glucan, and energy pathways, in both omics analyses (Fig. 2B and C). Moreover, the cellular response to starvation was elevated in response to acute HEV infection, suggesting that AMPK activation occurs in response to the deficient energy caused by decreased nutrient metabolism in the host.

We next sought to characterize whether the energy alterations described above were associated with alterations in mitochondrial morphology and function. Consistent with increased energetic stress, TEM revealed severe disruption of the mitochondrial ultrastructure, with some mitochondria losing cristae (Fig. 2D). In parallel, pathway enrichment analysis revealed that the oxidative damage response was enhanced in acute HEV infection (Fig. 2E). However, the mitochondrial membrane potential (MMP) was not much different in the acute HEV model (Fig. S3A). These results show that HEV is associated with substantial mitochondrial ultrastructural remodeling, characterized by reduced cristae and oxidative damage.

Excessive reactive oxygen species (ROS) production can lead to macromolecule oxidation with free radical attack of phospholipids, disturbing the integrity of mitochondrial membranes, mitochondrial membrane depolarization, and mtDNA mutation [27]. We measured the levels of ROS to clarify whether mitochondrial structural damage induced by HEV particles is related to ROS. We found that ROS levels were lower in acute HEV infection (Fig. S3B). Excess ROS or RNS are typically associated with increased oxidative damage [28]. Our data indicate that the mitochondria predominantly maintain energy balance, an event that is compromised by oxidative damage in the presence of HEV. This disruption results in AMPK activation and a distortion of metabolism that is independent of ROS levels.

### AMPK activation suppressed HEV replication

**Fig. 3:**
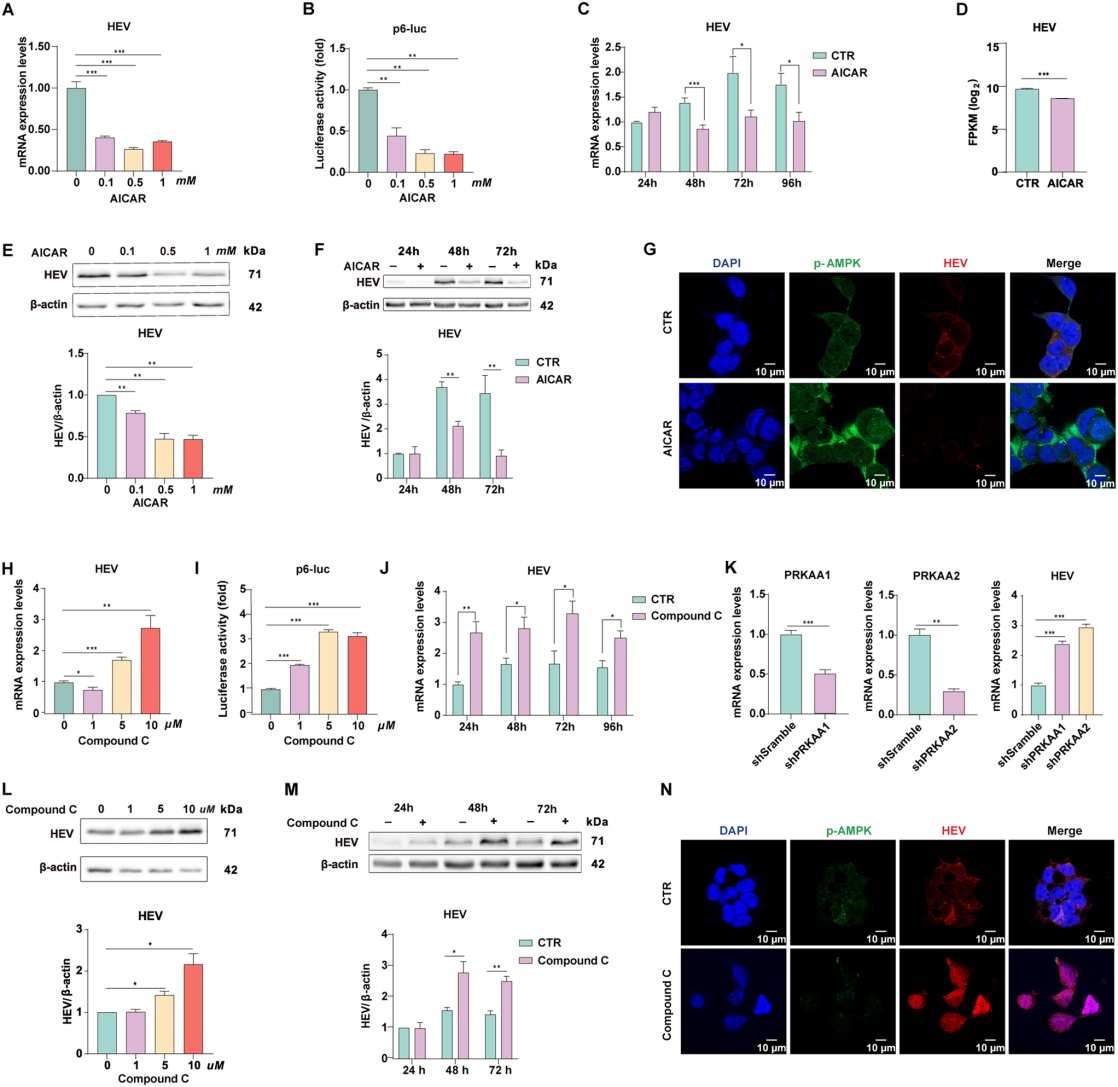
Activation of AMPK suppressed HEV replication. P6 (**A**) and p6 luciferase (**B**) cells were treated with AICAR at different concentrations for 72 h. (**C**) P6 cells were treated with 1 mM AICAR for different durations. (**D**) RNA-Seq analysis was performed to determine the FPKM values of HEV sequences in control and P6 cells. (**E**-**F**) HEV protein levels were quantified in samples that received the same treatments as those in (**A**) and (**C**). (**G**) p-AMPK and HEV-ORF2 were costained and detected by immunofluorescence. (**H**) P6 and (**I**) P6 luciferase cells were treated with compound C at different concentrations for 72 h. (**J**) P6 cells were treated with 10 µM compound C at different time points. (**K**) P6 cells were stably integrated with lentiviral shRNAs targeting the scramble sequence, *PRKAA1* or *PRKAA2*. (**L**-**M**) HEV protein levels were quantified in samples that received the same treatments as those in (**H**) and (**I**). (**N**) p-AMPK and HEV (ORF2) were costained and detected by immunofluorescence. The data were normalized to those of the control group (CTR, set as 1). The data represent at least three experiments and are presented as the mean ± SEM. *P < 0.05; **P < 0.01; ***P < 0.001

AICAR is an AMPK agonist. To determine the role of AMPKα in HEV replication, we tested the effects of 0, 0.1, 0.5 and 1 mM AICAR on viral replication via a the full-length (p6) infection model. These results showed that active AMPK significantly inhibited cellular viral RNA replication in the chronic infectious HEV model in a dose-dependent manner (Fig. 3A). To confirm this finding, we used an HEV replicon model in which cells were transfected with a subgenomic construct of HEV with the in-frame secreted form of luciferase [29]. The accumulation of luciferase serves as a reporter for HEV RNA synthesis (p6-luc). Correspondingly, treatment with AICAR resulted in a significant reduction in HEV replication-related luciferase activity in the subgenomic replicon. (Fig. 3B). Furthermore, the antiviral activity of AICAR against HEV was also time-dependent. Compared with the control, treatment with AICAR at the 48th, 72nd and 96th h post treatment resulted in 38.5%, 44.5% and 42.2% lower HEV genomic RNA levels, respectively (as determined by RT‒qPCR) (Fig. 3C). The FPKM value of HEV was also decreased in p6 cells treated with AICAR according to RNA-seq analysis (Fig. 3D). Furthermore, immunoblot analysis revealed that the level of the HEV ORF2 protein was markedly decreased in a dose- and time-dependent manner (Fig. 3E and F). A suppressive effect of AMPK on HEV was also observed by confocal microscopy (Fig. 3G).

Accordingly, compound C (an AMPK inhibitor) resulted in 1.74-fold and 2.30-fold increases in HEV replication at most in both the p6 and p6-luc models (Fig. 3H and J), further confirming that AMPK activity negatively regulates HEV replication. Consistently, western blot and immunofluorescence assays demonstrated the pro-HEV activity of the AMPK inhibitor at the post translational level (Fig. 3L and N). In addition to pharmacological inhibitors, lentiviral-mediated RNA interference was used for the knockdown of *PRKAA1* and *PRKAA2*, allowing the investigation of the direct function of AMPK in HEV replication. P6 cell models are stably integrated with shRNAs targeting the scramble sequence (as a control), *PRKAA1* or *PRKAA2*. The cellular HEV genomic viral RNA was subsequently quantified. The silencing of *PRKAA1* resulted in a 1.37-fold (*P* < 0.001) increase in HEV RNA, and the silencing of *PRKAA*2 resulted in a 1.93-fold (*P* < 0.001) increase in viral genomic RNA (Fig. 3K). Collectively, these data demonstrated that AMPKα is a host antiviral factor that protects against HEV replication.

### AMPK activation mediated the HEV-induced antiviral immune response

**Fig. 4:**
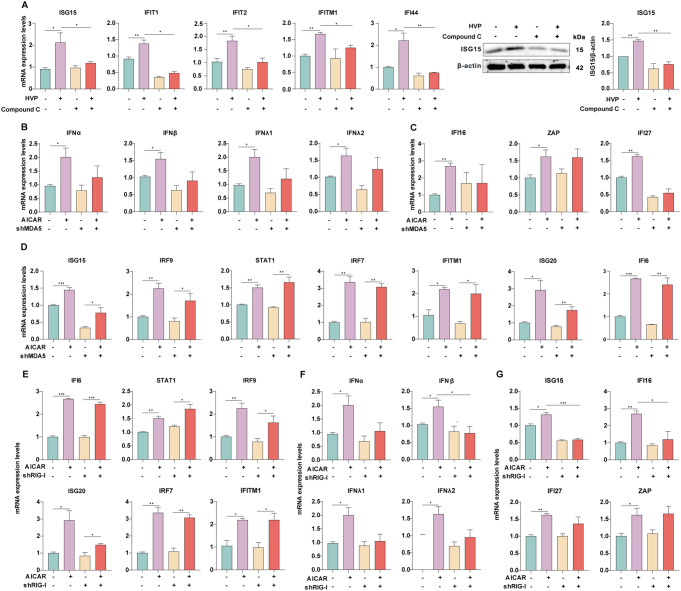
AMPK activation mediated the HEV-induced antiviral immune response (**A**) Huh7 cells, with or without HEV infection, were treated with Compound C for 48 h. (**B**) Type I and III IFNs and (**C**-**D**) ISGs were detected in *MDA5*-deficient Huh7 cells following AICAR treatment. (**E**-**G**) ISGs and type I and type III IFNs were detected in *RIG*-I-deficient Huh7 cells following AICAR treatment. The data were normalized to those of the control group (CTR, set as 1). The data represent at least three experiments and are presented as the mean ± SEM. *P < 0.05; **P < 0.01; ***P < 0.001

We previously reported that HEV infection can potently induce IFN production and antiviral responses in various cell and organoid models, as well as in patients [30]. Moreover, a previous study demonstrated that activation of AMPK enhances the expression of ZIKV-induced IFNs and their downstream effectors, ISGs [10]. RT‒qPCR confirmed that IFNs and ISGs were also stimulated when AMPK was activated by AICAR (Fig. S4A). These findings prompted us to investigate whether AMPK mediated HEV-induced IFN gene expression. Figure 4 A shows that pharmacological suppression of AMPK completely blocked the stimulation of a subset of ISGs triggered by HEV, suggesting that AMPK is required for antiviral ISG induction upon HEV infection.

RIG-I and melanoma differentiation-associated protein 5 (MDA5) are two members of the RLR family that are ubiquitously expressed to mediate the antiviral response to RNA viruses. Our previous study demonstrated that the overexpression of MDA5 or RIG-I has anti-HEV effects; however, the two RLRs are not essential for eliciting an IFN response to HEV infection [31, 32]. We, therefore, asked whether RIG-I and MDA5 are implicated in AMPK-mediated ISG activation in the context of HEV infection. We first suppressed MDA5 with lentiviral shRNA, which was confirmed by RT‒qPCR and western blot assays (Fig. S5A and S5B). Interestingly, deficiency of MDA5 profoundly attenuated both type I (IFNα and IFNβ) and type III IFN (IFNλ1 and IFNλ2) transcription, suggesting that the AMPK-triggered IFN response imperatively requires MDA5 (Fig. 4B). To elucidate further whether MDA5 is also necessary for downstream ISG induction, we compared the expression profiles of a subset of ISGs after AICAR treatment between MDA5 silenced and control cells. Strikingly, among the ten tested AICAR-induced ISGs, seven ISGs were not affected at all and could still be facilitated by AICAR in MDA5-knockdown cells (Fig. 4C and D). These results suggest that AMPK-stimulated ISGs are mostly independent of MDA5 and even independent of IFN production. A similar response was also observed in cells upon the silencing of RIG-1 (Fig. S5C-S5D and 4E-4G). Collectively, these data suggest that MDA5 and RIG-I are essential for AMPK-induced IFN expression but are not necessary for the majority of AMPK-induced ISGs. IFN-independent ISG activation might contribute to mediating the AMPK-induced antiviral immune response.

### AMPK-mediated ISG induction is independent of the JAK-STAT pathway

**Fig. 5:**
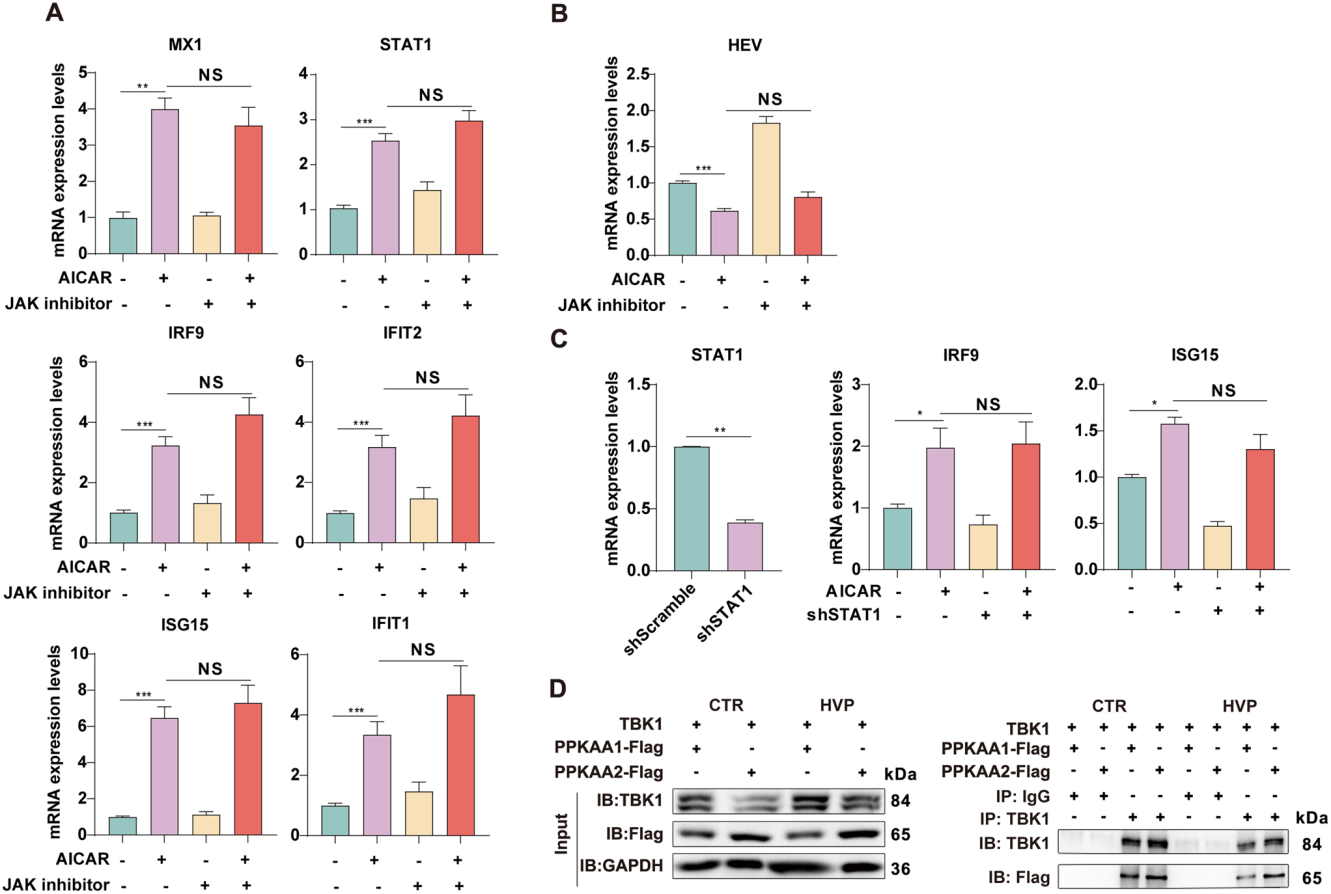
AMPK restricted HEV replication and ISGs induction regardless of JAK-STAT pathway activation (**A**-**B**) P6 cells were treated with 1 mM AICAR, with or without 10 µM Pyridone 6 for 48 h. (**C**) ISGs were detected in STAT1-deficient Huh7 cells following AICAR treatment. (**D**) Huh7 cells were cotransfected with TBK1 and PRKAA1-flag/ PRKAA2-flag and then infected with HEV for 48 h. The data were normalized to those of the control group (CTR, set as 1). The data represent at least three experiments and are presented as the mean ± SEM. *P < 0.05; **P < 0.01; ***P < 0.001

Given that the JAK-STAT cascade mediates the IFN response to induce ISG expression [2, 13], we then investigated whether ISG induction and the anti-HEV potency of p-AMPK are dependent on JAK-STAT activation. The JAK inhibitor, pyridone 6, was used to block the JAK-STAT pathway pharmacologically. As shown in Fig. S6A, IFN-stimulated ISGs were diminished by the JAK inhibitor. Accordingly, the anti-HEV activity of IFN was largely disrupted by this inhibitor (Fig. S6B), suggesting that HEV replication was negatively regulated by IFN-mediated ISGs via the JAK-STAT1 pathway. However, the AMPK-induced activation of ISGs was not affected by the JAK inhibitor, suggesting that JAK-STAT is not required for AMPK -induced antiviral innate immunity (Fig. 5A). Moreover, AMPK retained profound anti-HEV potency in the presence of the JAK inhibitor (Fig. 5B). To further confirm that AMPK -induced ISGs are not dependent on the JAK-STAT pathway, we stably knocked down STAT1 in Huh7 cells. Like the JAK inhibitor, the suppression of STAT1 did not abrogate ISG production in response to the AMPK activator (Fig. 5C). These data suggest that AMPK restricts HEV replication and ISGs induction regardless of JAK-STAT pathway activation. TBK1 activation facilitates IRF3 phosphorylation at the C-terminus via the MAVS or STING signalosome to transcribe type I interferons (IFN-Is) and numerous IFN-stimulated genes (ISGs) in response to invasion and damage, both directly and indirectly. Moreover, a previous study revealed that TBK1 is modified at a classic substrate motif by AMPK [14]. We postulated that AMPK activation regulates antiviral innate immune responses by activating TBK1. As anticipated, coimmunoprecipitation revealed an interaction between cotransfected TBK1 and the AMPKα subunits (PRKAA1 and PRKAA2) and a greater level of stably expressed PRKKA2 with TBK1 in Huh7 cells, particularly upon HEV infection (Fig. 5D), suggesting that HEV facilitates the interaction of TBK1 and the AMPK subunit, which might contribute to downstream ISG transcription.

### AMPK activation inhibited HEV replication by reducing the number of autophagosomes

**Fig. 6:**
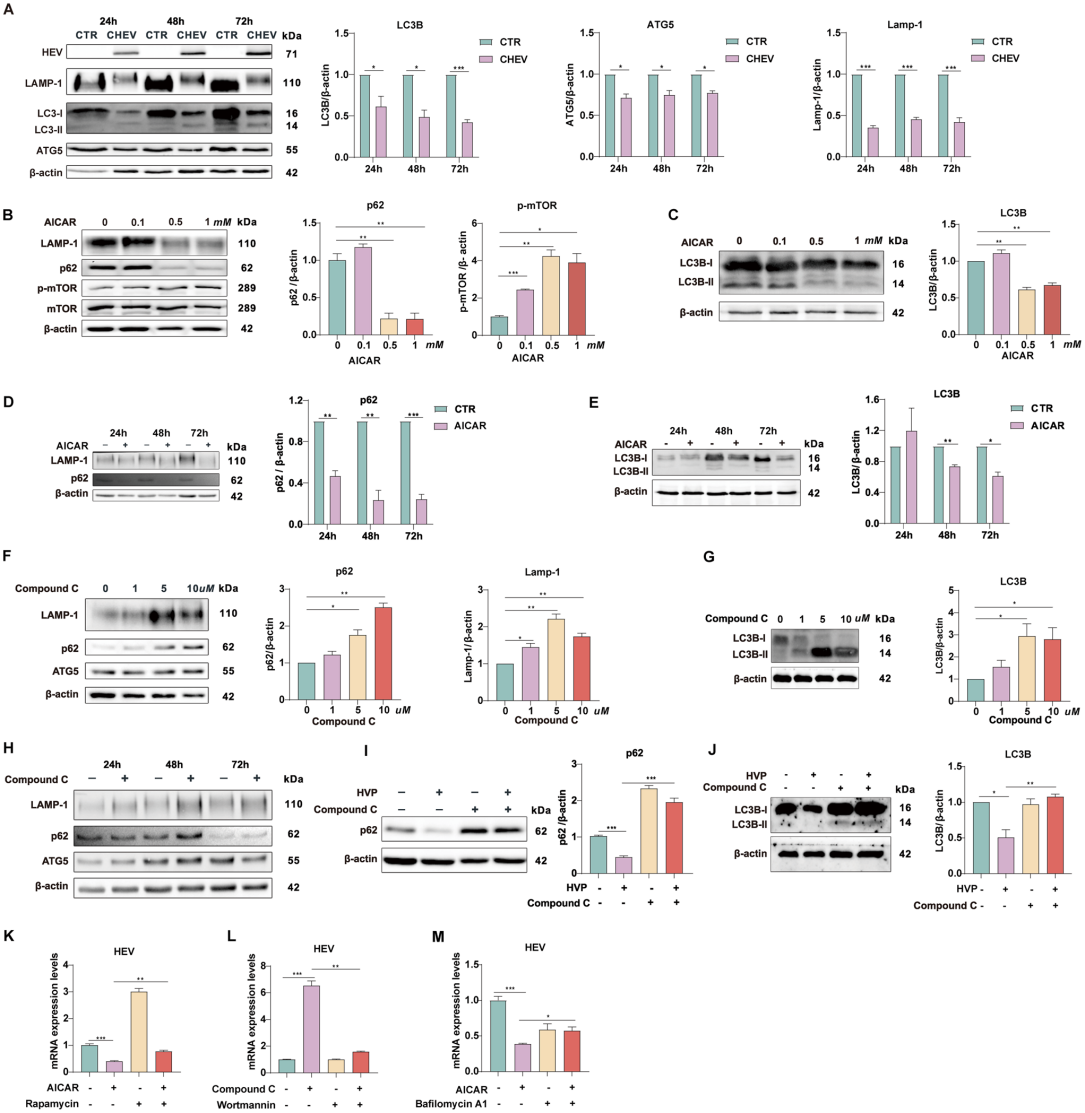
AMPK activation inhibited HEV replication by reducing the number of autophagosomes. (**A**) P6 cells were harvested to measure the autophagy indicators. (**B**-**C**) The protein levels of LAMP-1, p62, p-mTOR, mTOR, LC3B-I and LC3B-II were detected in Huh7 cells treated with AICAR at different concentrations for 48 h. (**D**-**E**) Protein levels of LAMP-1, p62, LC3B-I and LC3B-II were detected in Huh7 cells treated with 1 mM AICAR for different durations. (**F**-**G**) Protein levels of LAMP-1, p62, ATG5, LC3B-I and LC3B-II were detected in Huh7 cells treated with different concentrations of compound C for 48 h. (**H**) Protein levels of LAMP-1, p62 and ATG5 were detected in Huh7 cells treated with 10 µM compound C for different durations. (**I**-**J**) Protein levels of p62, LC3B-I and LC3B-II were detected in Huh7 cells upon HEV infection with Compound C. (**K**) Huh7 cells were treated with 1 mM AICAR and 1 µM rapamycin, (**L**) 50 nM wortmannin, or (**M**) 100 nM bafiloycin for 48 h. The data represent at least three experiments and are presented as the mean ± SEM. *P < 0.05; **P < 0.01; ***P < 0.001

Autophagy is an important cellular factor that many viruses utilize for replication [20–22]. AMPK is reported to be a key regulator of autophagy in response to various stresses [23]. Therefore, we explored whether autophagy-mediated the inhibition of HEV caused by AMPK. We first determined the effect of HEV on the autophagic process. Compared with control cells, P6 cells presented significantly lower levels of Lamp-1, ATG5 and LC3 (Fig. 6A), suggesting that HEV blocks the early stages of autophagy but induces autophagic degradation, leading to a decrease in the number of autophagosomes. We then determined the role of AMPK in autophagy. Immunoblot analysis of Huh7 cells treated with AICAR revealed an increase in p-mTOR and the inhibition of Lamp-1, p62, and LC3 (Fig. 6B and E). Conversely, Huh7 cells treated with the AMPK inhibitor Compound C presented increased expression of ATG, p62, lamp-1 and LC3 (Fig. 6F and H). These results collectively suggest that activated AMPK inhibits the accumulation of autophagosomes by regulating the induction of autophagy and autophagic degradation. Given that both HEV and AMPK can negatively regulate autophagosome accumulation, we subsequently elucidated whether AMPK is the critical mediator involved in HEV mediated suppression of autophagy. Figure 6I and J shows that pharmacological inhibition of AMPK rescued p62 and LC3 production inhibited by HEV infection, indicating that AMPK is indispensable for HEV-induced suppression of autophagosome accumulation.

Considering that AMPK activation regulates both the autophagic process and HEV replication, autophagosomes may be involved in AMPK-regulated HEV replication. We observed that the activation of autophagosome formation by rapamycin reversed the inhibition of HEV replication by AICAR (Fig. 6K). Consistently, the inhibition of autophagy by wortmannin (an inhibitor of the early stages of autophagy) abrogated the pro-viral effect of the AMPK inhibitor (Fig. 6L). To further investigate the role of autophagosomes in the anti-HEV effect of AMPK, we used bafilomycin A1 to suppress the autophagic degradation process. As expected, the suppression of autophagosome degradation abolished the anti-HEV effect of AMPK activation (Fig. 6M). These results demonstrated that a reduced number of autophagosomes was largely responsible for maintaining the antiviral effect of p-AMPK on HEV.

### AMPK activation suppressed HEV-induced inflammation

**Fig. 7:**
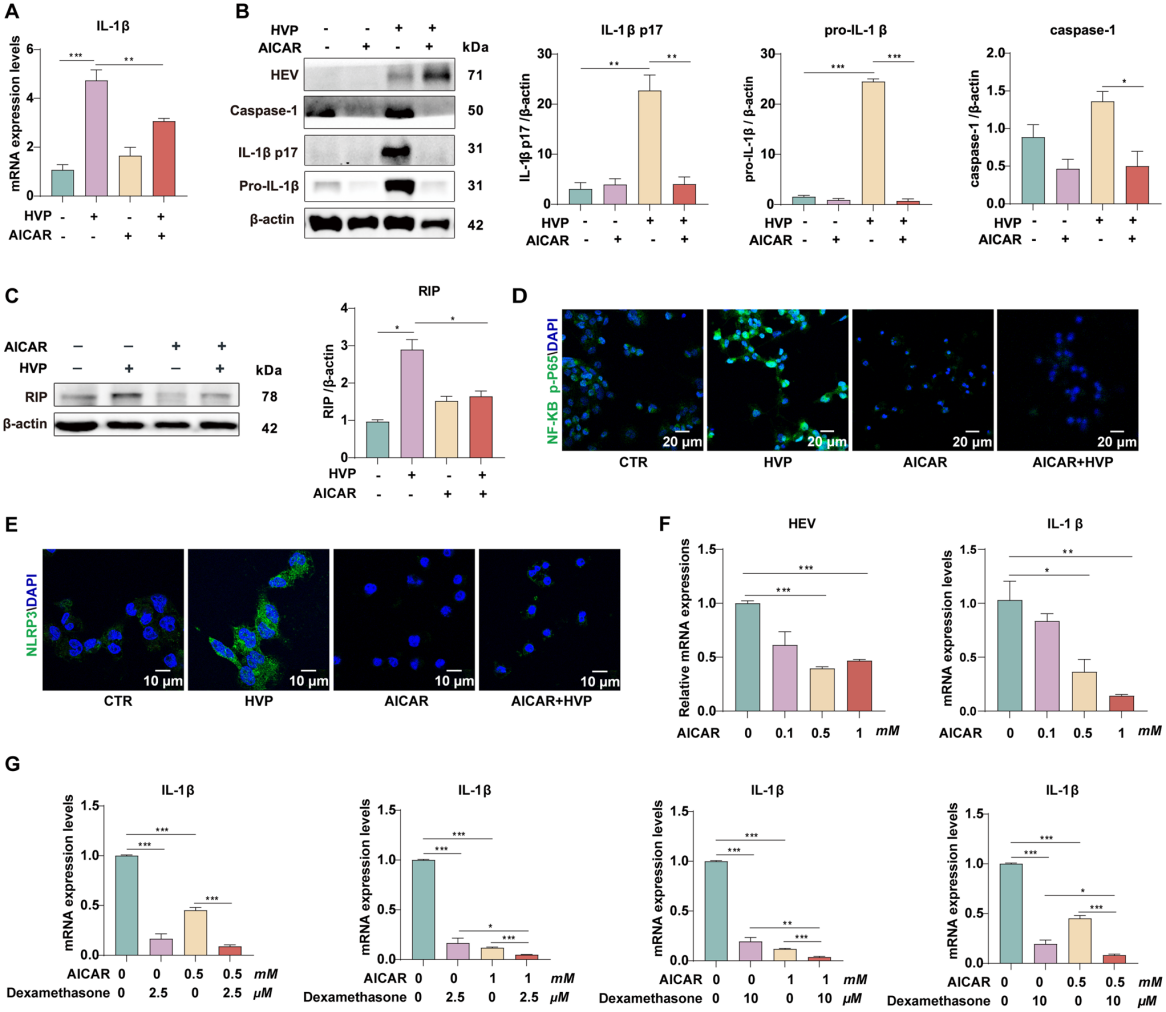
AMPK activation suppressed the HEV-induced inflammatory response. (**A**-**C**) Differentiated THP-1 cells infected with HEV were treated with AICAR for 48 h. (**D**-**E**) NF-κB p-P65 and NLRP3 were detected via immunofluorescence. (**F**) P6 cells were coculture with THP-1 macrophages. This coculture system was treated with AICAR at different concentrations for 48 h. (**G**) Huh7 cells were cocultured with THP-1 macrophages. This system was treated with AICAR and dexamethasone at different concentrations for 48 h. The data were normalized to those of the control group (CTR, set as 1). The data represent at least three experiments and are presented as the mean ± SEM. *P < 0.05; **P < 0.01; ***P < 0.001

We previously demonstrated reciprocal antagonism between the HEV-induced inflammasome and the IFN response [2]. Given that AMPK activation stimulates IFNs and ISGs, we next investigated the effects of targeting AMPK on the inflammatory response induced by HEV infection. We inoculated differentiated THP-1 cells with HEV particles in combination with AICAR. Figure 7A shows that HEV potently stimulated IL-1b, which was largely alleviated by treatment with AICAR. Western blotting confirmed the activation of IL-1β and caspase-1 upon HEV infection (Fig. 7B), which was blocked by AICAR treatment. Generally, IL-1β activation is regulated by two signals, including pro-IL-1β mRNA transcription regulated by NF-κB and the processing of IL-1β mediated by the NLRP3 inflammasome [2]. A previous study reported a crucial role for RIP1 in NF-κB activation [33]. Importantly, we observed that RIP was triggered by HEV (Fig. 7C). This activation by HEV was profoundly abolished by AICAR treatment (Fig. 7C). Accordingly, the translocation of the phosphorylated p65 subunit of NF-κB into the nucleus of Huh 7 cells and the increase in NLRP3 triggered by HEV infection was also inhibited by AICAR, as shown by immunofluorescence analysis (Fig. 7D and E). These results demonstrated that AMPK activation suppressed the inflammatory response triggered by HEV infection.

Monocytes and macrophages are the key drivers of pathological inflammation. Our previous study reported that HEV is capable of infecting human macrophages [2]. Given that hepatocytes are the primary targets of HEV infection, we established a coculture system of Huh7/p6 cells with THP-1 macrophages to assess the synergetic anti-inflammatory and antiviral effects of targeting AMPK. We found that AICAR significantly inhibited HEV replication and IL-1β expression simultaneously (Fig. 7F). Ribavirin, an antiviral agent employed against a spectrum of viruses, including hepatitis C virus (HCV) and coxsackievirus B3, has been demonstrated to inhibit HEV replication [34–36]. The anti-HEV effect of IFNa has also been reported, albeit not as a standard treatment modality. We then tested the combined anti-HEV activity of AICAR and ribavirin or IFNa. However, no synergetic anti-HEV effect was observed for any combination (Fig. S7A and S7B). Additionally, our previous study demonstrated that dexamethasone significantly inhibited the HEV-induced inflammasome response [2]. Therefore, we aimed to assess the combined anti-inflammatory effect of AICAR. In Huh7 with THP-1 cell coculture system treated with HEV particles, the combination of dexamethasone and AICAR simultaneously inhibited HEV-induced IL-1β production (Fig. 7G).

## Discussion

**Fig. 8:**
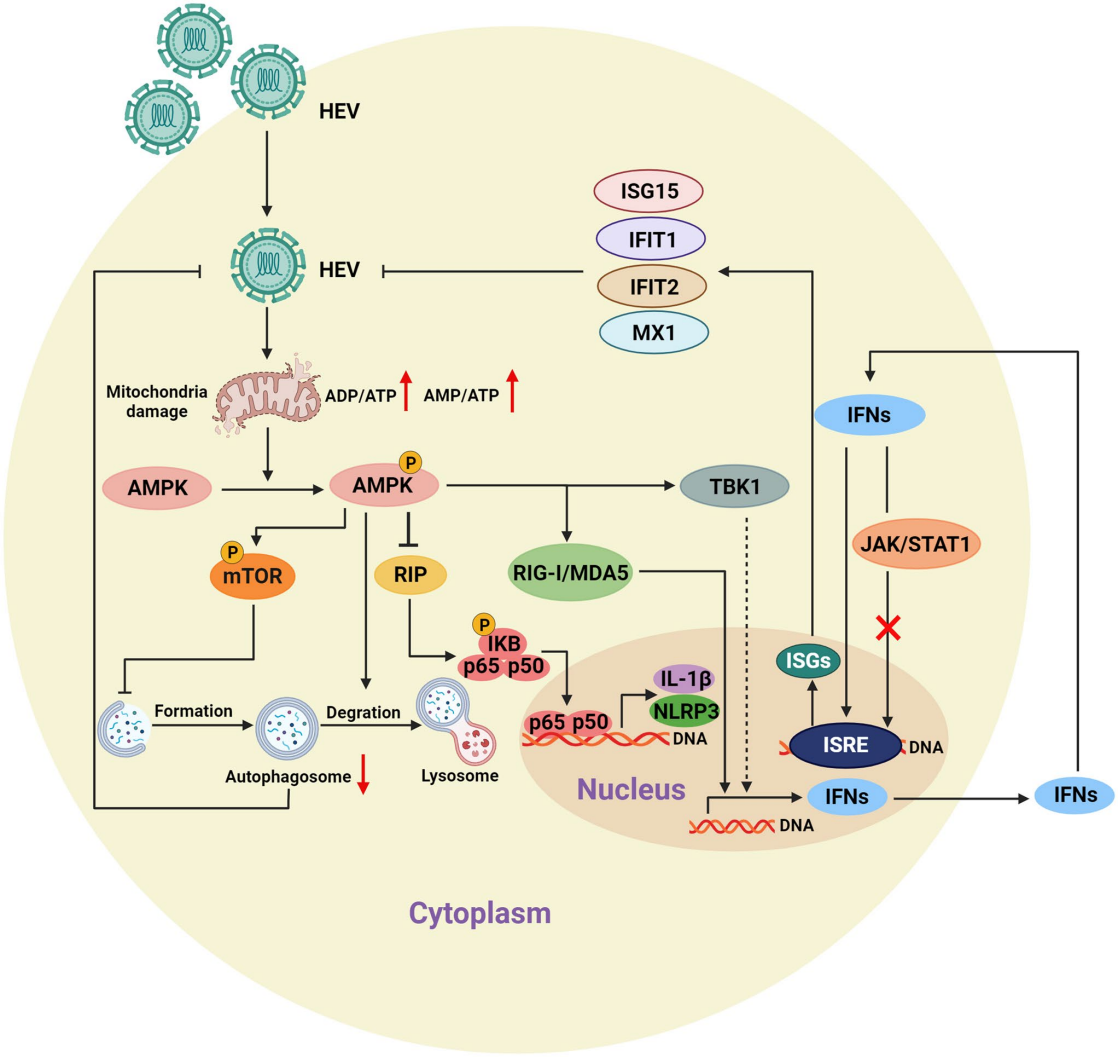
Working model of the study. Schematic representation of the mechanisms underlying the anti-inflammatory and antiviral effects of AMPK on HEV

Viruses generally rely on host cells for energy supplementation to support their replication [37, 38]. Understanding the metabolic changes in viruses may reveal novel therapeutic targets for combating viral diseases [39]. In this study, we found that HEV infection, either acutely or chronically, could potently activate the key metabolic sensor AMPK, which in turn suppressed HEV replication. Mechanistically, HEV injuries mitochondria, interfering with energy generation. AMPK, which monitors the cellular energy status, is activated by increased ratios of AMP: ATP and ADP: ATP during HEV infection. On the one hand, AMPK activation directly reinforces the HEV-induced innate immune response by combining with TBK1; on the other hand, it contributes to HEV-suppressed autophagosomes, resulting in the suppression of HEV replication. Moreover, activated AMPK attenuated the inflammatory response in HEV-infected macrophages. Accordingly, we demonstrated the dual therapeutic potential of hepatitis E through anti-inflammatory and antiviral activity through the agitation of AMPK (Fig. 8).

AMPK is a key metabolic sensor and regulator that is activated by various viral infections. This process is complex and context-dependent, as different viruses may exploit AMPK signaling in distinct patterns, facilitating or impairing their replication. Previous studies have demonstrated that AMPK activation has pro-viral effects on HCMV, DENV, and PEDV [40–44]. HCMV activates AMPK by promoting CAMKK to promote a glycolytic activation conducive to viral replication [43]. DENV has been reported to activate the AMPK-mTOR axis to stimulate pro-viral lipophagy [42]. PEDV infection activates AMPK and JNK through TAK1 to induce autophagy and enhance virus replication [44]. In contrast, some viruses, such as HSV-1, VSV, HBV, Zika virus and HCV, are able to promote diverse cellular antiviral responses via AMPK activation, leading to antiviral outcomes [10, 14, 45–47]. AICAR administration to BALB/c mice with corneal HSV-1 infection significantly improved AMPK-TBK1-mediated innate antiviral immunity against HSV-1, with evident amelioration of eyelid swelling, eye closure, and eye crusting [14]. Compared with VSV-infected WT mice, VSV-infected mice with conditional knockout of AMPKα1/α2 in myeloid cells presented somewhat reduced antiviral responses and increased viral replication via the phosphorylation of TBK1. AMPK activation in response to HBV-induced oxidative stress decreases HBV production through the promotion of autophagic degradation [45]. Similarly, AMPK signaling in endothelial cells reduces ZIKV infection by inhibiting glycolysis and enhancing innate antiviral responses [10]. Activated AMPK also inhibited HCV RNA replication by activating type I interferon signaling [46, 47]. One possible explanation underlying the differential effects of AMPK activation on viral infection is that viruses may activate AMPK through distinct pathways or at different stages of infection, leading to the phosphorylation of different AMPK substrates or downstream targets. Alternatively, different cell types may express different AMPK isoforms or cofactors, which could modulate the specificity or affinity of AMPK for its substrates or targets in response to viral infection.

In the present study, we found that the activation of AMPK by AICAR significantly potentiated the expression of IFNs and ISGs upon HEV infection. AMPK has been implicated in various host antiviral defences, specifically through regulating innate immune signaling via molecules such as STING, MAVS, IRF3, and TBK1 [8, 14, 48–50]. These results reveal a new regulatory circuit that serves as an important component of the host defence against virus infection. Interestingly, in our study, AMPK activation increased ISG expression independently of the activated JAK-STAT1 pathway, which was explained by recent studies that have shown that the activation of AMPK can limit JAK-STAT-dependent signaling pathways [51, 52].

One of the most intriguing findings is that HEV infection increases the binding of TBK1 to PRKAA2, the catalytic subunit of AMPK. TBK1 may subsequently contribute to IRF3 activation and IFN production [53]. A recent study reported that AMPK is able to directly phosphorylate TBK1 at Ser511, which enhances the binding of TBK1 to IRF3 with high affinity [14]. These results reveal a novel mechanism by which AMPK regulates innate immunity and antiviral defence through the modulation of TBK1 activity and function.

AMPK is a key regulator of autophagy during virus infection via specific virus-host interactions. In our study, we observed that the activation of AMPK prevented the initiation of autophagy and facilitated autophagic degradation, resulting in decreased autophagosome accumulation and HEV replication. This finding was supported by a previous report that AMPK inhibition was associated with impaired autophagic proteolysis [23]. Similarly, the activation of AMPK induced by HBV promotes the autophagic degradation pathway in hepatocytes, leading to a decrease in autophagosome accumulation and HBV replication [20, 21, 45, 54]. Conversely, AMPK activation is also able to induce autophagosome accumulation by triggering autophagy via ERK/mTOR signaling during DENV infection [55–57]. The inhibition of autophagic initiation significantly inhibited DENV replication [57–59], indicating a pro-viral effect of AMPK activation on DENV via autophagosome accumulation. Therefore, although the level of autophagosomes is diversely regulated by AMPK in response to distinct viruses, viral replication is positively correlated with autophagosomes regardless of AMPK activation.

In addition to its anti-HEV activity, AMPK also has powerful anti-inflammatory properties in HEV-infected macrophages. A previous study reported that AMPK activation by reduced choline uptake contributes to the termination of the LPS- and ATP-induced NLRP3 inflammasome and IL-1β production by stimulating mitophagy, which decreases the number of damaged mitochondria that produce oxidized- (ox) mtDNA [60]. Considering that the anti-inflammatory activity of AMPK is independent of that of glucocorticoids through their immunosuppressive effects on multiple signaling pathways, an intriguing question is whether AMPK activators can promote the anti-inflammatory efficacy of glucocorticoids. In our study, the combination of AICAR and dexamethasone displayed a general beneficial effect on the remission of inflammation, and no negative drug-drug interference was observed. This finding provides a proof of concept that combinations of different target agents constitute compatible functions against inflammatory diseases.

In conclusion, HEV infection activated AMPK, which regulates the innate antiviral response and autophagy to inhibit viral replication collectively. Importantly, pharmacological activation of AMPK simultaneously inhibits HEV infection and the inflammatory response. In addition, this therapeutic effect was further augmented when AICAR was combined with another anti-inflammatory regimen, dexamethasone. Future studies should focus on evaluating the antiviral effects of AMPK against HEV in vivo models. These results offer an attractive, promising therapeutic option for severe hepatitis E, warranting further research in animal models and patients.

## Electronic supplementary material

Below is the link to the electronic supplementary material.

Supplementary Material 1

## Data Availability

The data supporting the findings of the current study are available from the corresponding author upon reasonable request.
