# Supplementary material for: AMPK activation by hepatitis E virus infection inhibits viral replication through attenuation of autophagosomes and promotion of innate immunity
Source: Cell Mol Life Sci. 2025 Mar 13;82(1):111. doi: 10.1007/s00018-025-05634-8 (PMC11904043; doi:10.1007/s00018-025-05634-8)
Supplement: Supplementary file 1 — Supplementary Material 1 [file 18_2025_5634_MOESM1_ESM.docx]

**Supplementary information to:**

**AMPK activation by hepatitis E virus infection inhibits viral replication through attenuating autophagosome and promoting innate immunity**

**Supplementary materials and methods:**

**Plasmids and reagents**

The plasmids that contain PRKAA1 (GenBank Accession Number: NM_006251.6), PRKAA2 (GenBank Accession Number: NM_006252.4), TBK1 (GenBank Accession Number: NM_013254), and the subgenomic and full-length GT3 HEV genome (Kernow-C1 P6 clone, GenBank Accession Number: JQ679013) were used.

The Polyethylenimine Linear (PEI) MW40000 Transfection reagent (40816ES03) was purchased from Yeasen (Shanghai, China). The Gaussia-Lumi™ Gaussia Luciferase Reporter Gene Assay Kit was obtained from Beyotime (Shanghai, China). AICAR (HY-13417, MCE), Ribavirin (HY-B0434, MCE) and Puromycin (P8230, Solarbio) were dissolved in PBS. Rapamycin (A8167, APExBIO), Bafilomycin A1 (A8627, APExBIO), Wortmannin (HY-10197, MCE), Dexamethasome (HY-14648, MCE), Compound C (B3252, APExBIO), 12-O-Tetradecanoylphorbol 13-acetate (PMA, HY-18739, MCE) and Pyridone 6 (HY-14435, MCE) were dissolved in DMSO. Antibodies including AMPK (ab131512), p-AMPK (ab23875), β-actin (ab8226), RIP (ab72139) were from ABCAM (Cambridge, MA, USA). TBK1 (3504), p-mTOR (5536s), mTOR (2983), IL-1β (D3U3E) (12703), Cleaved IL-1beta (Asp116) (D3A3Z) (83186s), Caspase-1 (3866) and Phospho-NF-κB p65 (3033s) were from Cell Signaling (Danvers, USA). Flag (AF2852) and IgG (A0201) were purchased from Beyotime (Shanghai, China). Lamp-1 (sc-20011) antibody was obtained from Santa Cruz Biotechnology (Santa Cruz, USA). LC3 (PM036) and p62 (M162-3) were from MBL (Beijing, China), NLRP3 antibody (19771-1-AP) was obtained from was from Proteintech (Rosemont, USA). Hepatitis E Monoclonal Antibody (MAB8002) was purchased from Merck/Millipore (Merck KGaA, Germany). Goat anti-Rabbit IgG (H+L) Highly Cross-Adsorbed Secondary Antibody, Alexa Fluor™ 488 (A-11034) and Goat anti-Mouse IgG (H+L) Highly Cross-Adsorbed Secondary Antibody, Alexa Fluor™ 555 (A-21424) were from Thermo Fisher Scientific (USA)

**Quantitative real-time PCR (qRT-PCR) analysis**

Total RNA was extracted from cultured cells using a high-purity total RNA extraction kit (BioTeke, China) according to the manufacturer’s instructions and was quantified with the Nanodrop 2000 (Thermo Fisher Scientific, USA). Complementary DNA (cDNA) was synthesized from 500 ng of RNA using a cDNA synthesis kit (TaKaRa, Japan). Relative gene expression levels were assessed using quantitative polymerase chain reaction (qPCR) with Hieff UNICON® advanced qPCR SYBR Master Mix (Yeasen). Human glyceraldehyde-3-phosphate dehydrogenase (GAPDH) was used as reference gene to normalize gene expression. Primer sequences used were shown in Supplementary information, Table S1.

**Western blotting analysis**

Cell lysates were prepared by incubating cells with ice-cold RIPA buffer (APExBIO) containing proteinase inhibitor cocktail and phosphatase inhibitor cocktail on ice for 30 min. The supernatant or lysates were then mixed with loading buffer and heated at 95°C for 5 min. Denatured proteins were equally loaded on SDS–PAGE gel, separated at 100 V for 100 min, and transferred to a PVDF membrane (pore size: 0.22 μm; Thermo Fisher Scientific Life Sciences) for 100 min with an electric current of 230 mA. Subsequently, the membrane was blocked with blocking buffer in PBS containing 0.05% Tween-20. Membranes were incubated with indicated primary antibodies overnight at 4°C. The membrane was washed 3 times, followed by incubation for 1 hour with corresponding HRP labelled secondary antibodies at room temperature, and washed five times. Protein bands were visualized using Western ECL Substrate. The membrane was scanned by Odyssey Infrared Imaging System (Li-Cor Biosciences). The bands were analysed and quantitated using Image J software. Relative protein levels were normalized to according β-actin controls.

**Gene Knockdown by Lentiviral Vector Delivered Short Hairpin RNA (shRNA)**

Short hairpin RNA (shRNA) sequences targeting PRKAA1, PRKAA2, MDA5, RIG-I and STAT1 were synthesized by Azenta life Sciences and inserted into pLKO.1 plasmid between the EcoRI and AgeI restriction sites. To generate stable gene knockdown cells, Huh7 cells were transfected by lentiviral vectors that also express a puromycin resistance gene. Transfected cells were subsequently selected by adding 2.5 µg/mL puromycin in the cell culture medium.

**Supplementary table S1**

Supplementary table S1. Primer sequences

| **Name** | **Sequences** |
| --- | --- |
| GAPDH-F | GAAGGTGAAGGTCGGAGTC |
| GAPDH-R | GAAGATGGTGATGGGATTTC |
| PRKAA2-F | TTCCTCAACACCTCAGCGTTC |
| PRKAA2-R | AAGACAATGTGCTTCCGGTCA |
| PRKAA1-F | CCCCATCCTGAAAGAGTACCAT |
| PRKAA1-R | ATGCCATTTTGCTTTCCTTACACC |
| CAMKK1-F | CACTGCCAGAAGATCGTCCAC |
| CAMKK1-R | GTTCCCCTCAAACTGGTTGCT |
| CAMKK2-F | TGCAGCTGAATCAGTATACCCT |
| CAMKK2-R | TGGACAGCACCTTCATTGCAT |
| STK11-F | CGTTCATCCACCGCATCGACT |
| STK11-R | CCTTCACCTTGCCGTAAGAGC |
| HEV-F | GACCGCTGAGCTTACTACCACA |
| HEV-R | ACCACCTAGAAGCGTATCAGCAA |
| ISG15-F | CTCTGAGCATCCTGGTGAGGAA |
| ISG15-R | AAGGTCAGCCAGAACAGGTCGT |
| IRF9-F | ACCGAAGTTCCAGGTAACACT |
| IRF9-R | CTCCAGCAAGTATCGGGCAA |
| MDA5-F | TCGAATGGGTATTCCACAGACG |
| MDA5-R | GTGGCGACTGTCCTCTGAA |
| IFITM1-F | AACCTTCACTCAACACTTCCTTC |
| IFITM1-R | TCCTCCTTGTGCATCTTC |
| ISG20-F | AACAGCCTGCTTGGACAC |
| ISG20-R | CGGATTCTCTGGGAGATTTGAT |
| IFI44-F | CAAGCTAGAGGAAGTCCAAAG |
| IFI44-R | CCACCGAGATGTCAGAAAG |
| IFIT1-F | GCCTTGCTGAAGTGTGGAGGAA |
| IFIT1-R | ATCCAGGCGATAGGCAGAGATC |
| IFIT2-F | GGAGCAGATTCTGAGGCTTTGC |
| IFIT2-R | GGATGAGGCTTCCAGACTCCAA |
| STAT1-F | ATGGCAGTCTGGCGGCTGAATT |
| STAT1-R | CCAAACCAGGCTGGCACAATTG |
| IFN-α-F | TGGGCTGTGATCTGCCTCAAAC |
| IFN-α-R | CAGCCTTTTGGAACTGGTTGCC |
| IFN-β-F | CTTGGATTCCTACAAAGAAGCAGC |
| IFN-β-R | TCCTCCTTCTGGAACTGCTGCA |
| IFN-λ1-F | GGAAGACAGGAGAGCTGCAACT |
| IFN-λ1-R | AACTGGGAAGGGCTGCCACATT |
| IFN-λ2-F | TCGCTTCTGCTGAAGGACTGCA |
| IFN-λ2-R | CCTCCAGAACCTTCAGCGTCAG |
| IL-1B-F | ATTTGAGTCTGCCCAGTTCCC |
| IL-1B-R | CAGTTATATCCTGGCCGCCTT |
| IRF7-F | AGCGCCAACAGCCTCTAT |
| IRF7-R | CAGCTTTCTGGAGTTCTCATTA |
| IFI6-F | GGTCTGCGATCCTGAATGGG |
| IFI6-R | TCACTATCGAGATACTTGTGGGT |
| IFI16-F | AGACTGAAGACTGAACCTGAAGA |
| IFI16-R | GAACCCATTGCGGCAAACATA |
| ZAP-F | CCGGTGCAACTATTCGCAGT |
| ZAP-R | TCAGTCCAGAGAGTTCGTGATTT |
| IFI27-F | CTGTCATTGCGAGGTTCTACT |
| IFI27-R | CCTGGCATGGTTCTCTTCT |
| RIG-I-F | AGAATCTGCAAAGACCTCGAA |
| RIG-I-R | CTGGCATCTGGAACACCAT |
| MX1-F | GGCTGTTTACCAGACTCCGACA |
| MX1-R | CACAAAGCCTGGCAGCTCTCTA |

**Supplementary Fig S1-Fig S7**

**
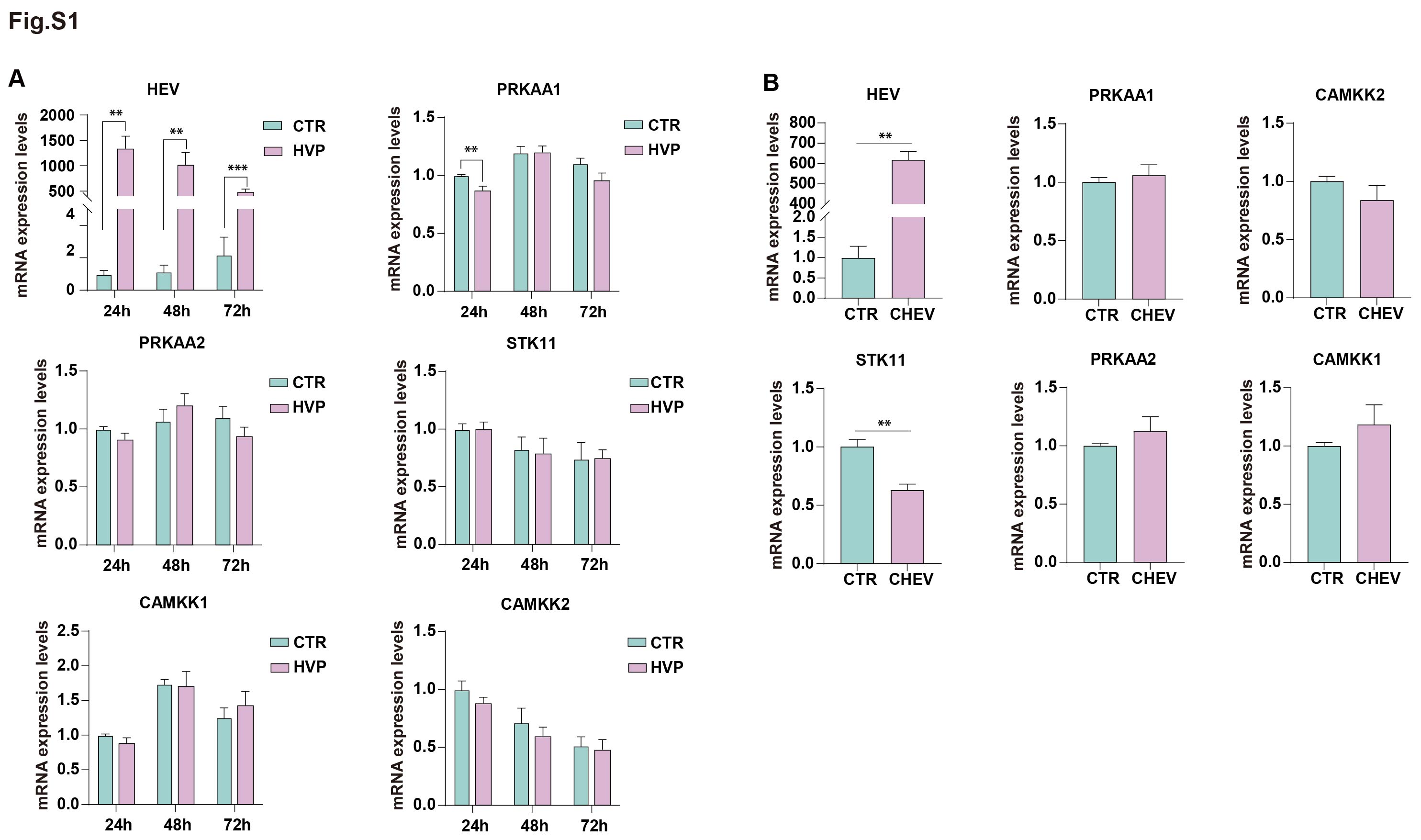
**

**Fig. S1 Transcriptional level of AMPKα and its upstream regulators were not affected in HEV-infected cells.** (A) Naïve Huh7 cells were inoculated with HEV viral particle, and mRNA levels of PRKAA1, PRKAA2 and their upstream regulatory genes were detected. (B) mRNA levels of PRKAA1, PRKAA2 and their upstream regulatory genes were quantified in chronic HEV infected Huh7 cells. The data represent at least three experiments and are presented as means ± SEM. **P* < 0.05; ***P* < 0.01; ****P* < 0.001.


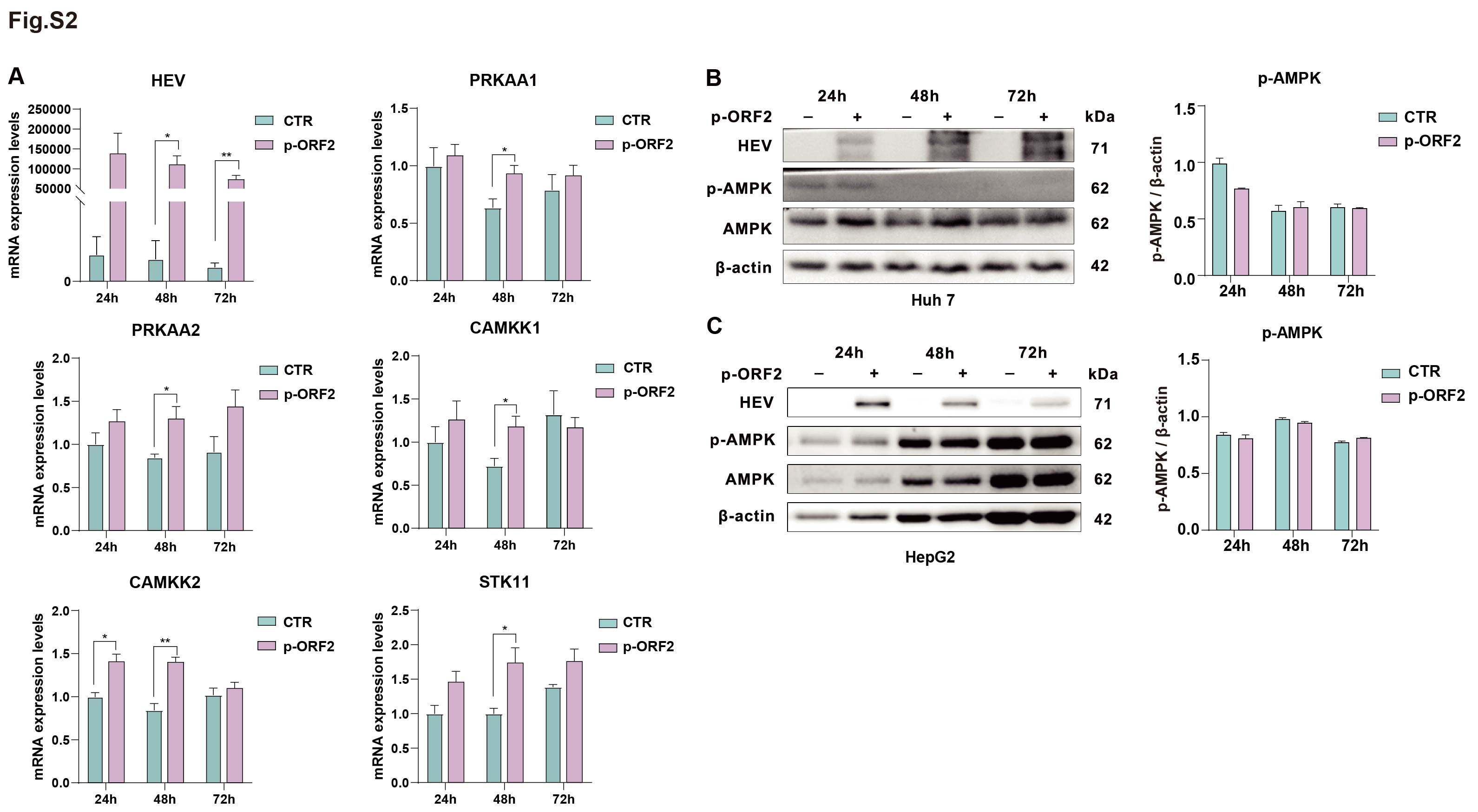


**Fig. S2 HEV ORF2 transfection did not affect the p-AMPK levels.** (A) Huh7 cells were transfected with plasmid expressing HEV ORF2. PRKAA1, PRKAA2 and their upstream regulated genes mRNA levels were detected. (B-C) Both Huh7 cells and HepG2 cells were transfected with the HEV ORF2 plasmid. Protein levels of HEV, p-AMPK and AMPK were quantified. The data represent at least three experiments and are presented as means ± SEM. **P* < 0.05; ***P* < 0.01; ****P* < 0.001.

**
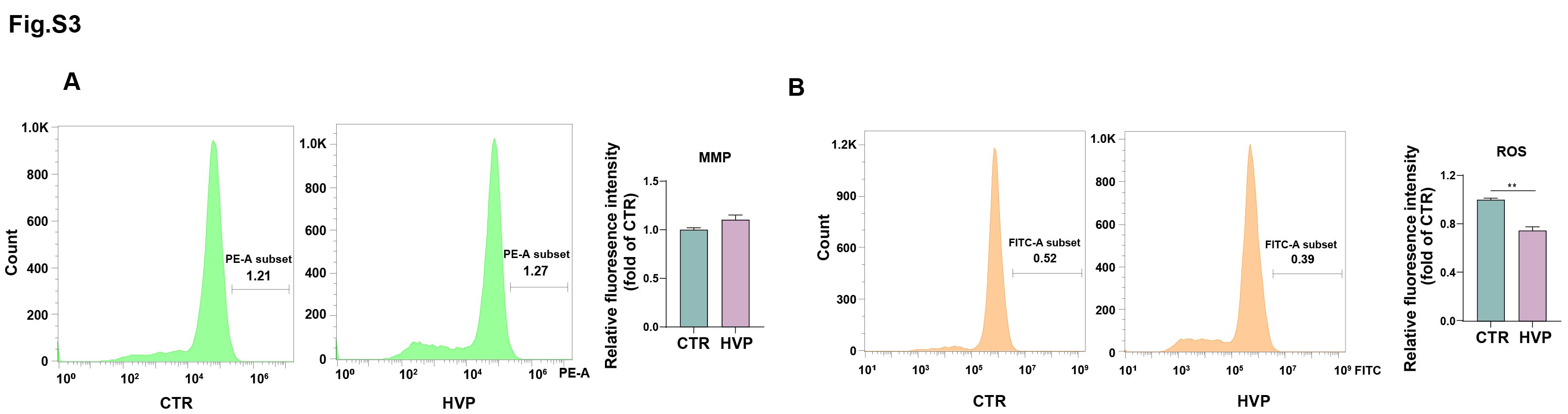
**

**Fig. S3 Neither ROS activation nor MMP alteration was observed in acute HEV infection**. Representative histograms of MMP (A) and ROS (B) from acute HEV model. Data were normalized to the control group (CTR, set as 1). The data represent at least three experiments and are presented as means ± SEM. **P* < 0.05; ***P* < 0.01; ****P* < 0.001.


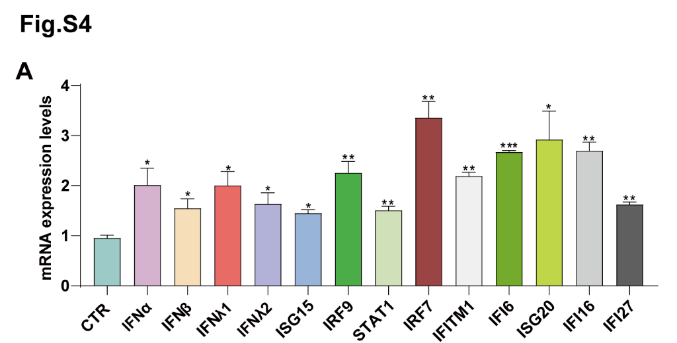


**Fig. S4 IFNs and ISGs were stimulated by activated AMPK.** (A)ISGs were determined when Huh 7 cells were treated with 1 mM AICAR for 48 hours. The data represent at least three experiments and are presented as means ± SEM. **P* < 0.05; ***P* < 0.01; ****P* < 0.001.


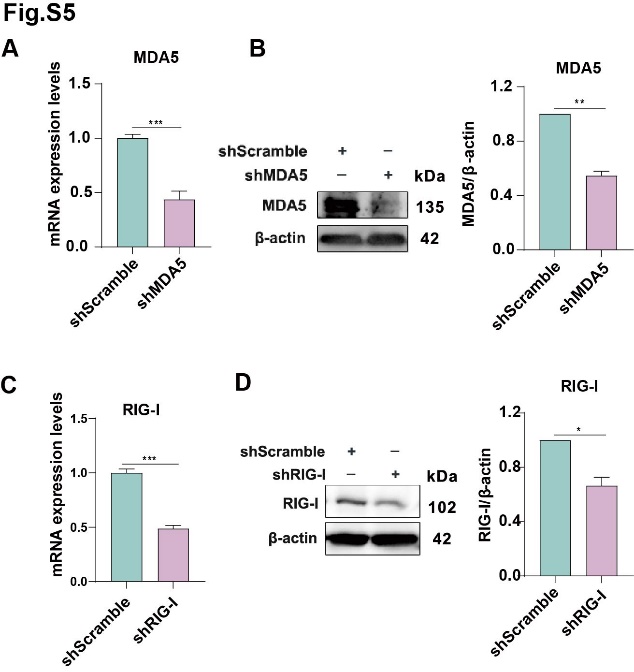


**Fig. S5 MDA5 and RIG-I were knocked down through stable integration of targeted lentiviral shRNA.** (A-B) MDA5 were suppressed in Huh7 cells. (C-D) RIG-I were suppressed in Huh7 cells. The data represent at least three experiments and are presented as means ± SEM. **P* < 0.05; ***P* < 0.01; ****P* < 0.001.


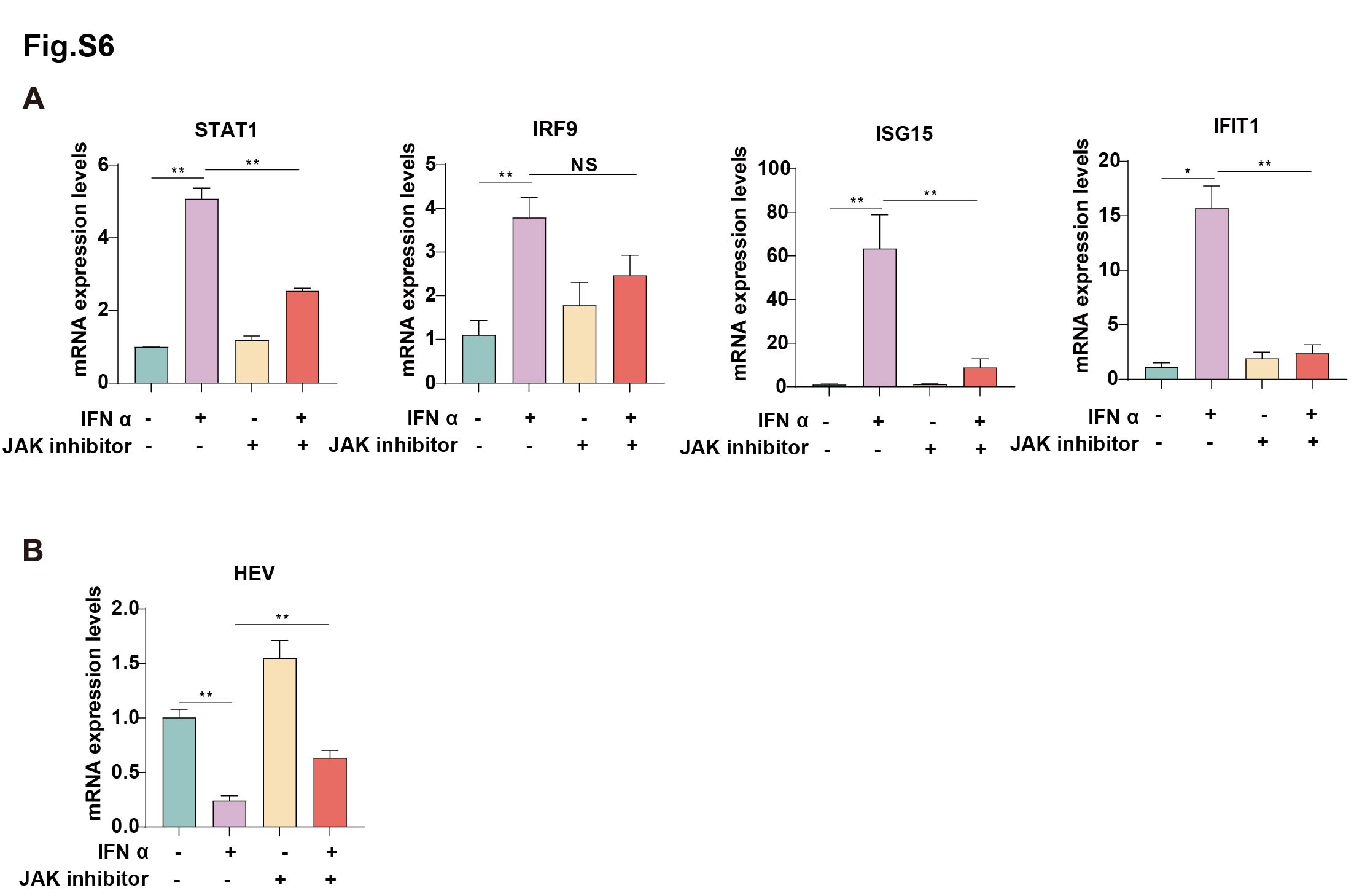


**Fig. S6 JAK mediates IFN-stimulated ISG expression, enhancing IFN's anti-HEV activity.** (A-B) P6 cells were treated with 10 ng/ml IFNα or/and 10μM Pyridone 6 for 48 hours. The data represent at least three experiments and are presented as means ± SEM. **P* < 0.05; ***P* < 0.01; ****P* < 0.001.


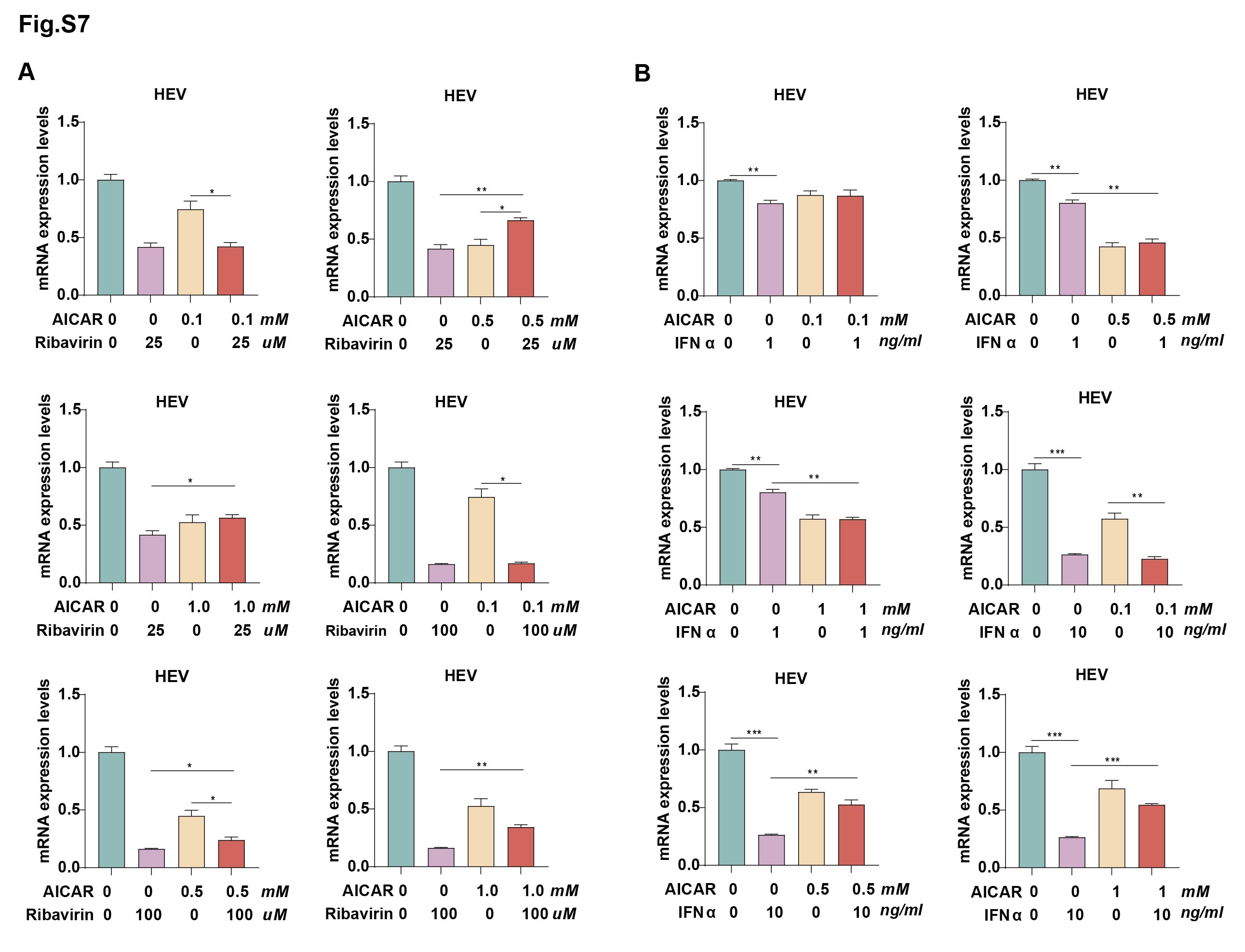


**Fig. S7 No synergistic anti-HEV effect was observed when AICAR was combined with ribavirin/ IFNα.** (A) P6 cells were treated with AICAR and Ribavirin or IFNα at different concentration for 48 hours. (B) P6 cells were treated with AICAR and IFNα at different concentration for 48 hours. The data represent at least three experiments and are presented as means ± SEM. **P* < 0.05; ***P* < 0.01; ****P* < 0.001.
